# Airway disease decreases the therapeutic potential of epithelial stem cells

**DOI:** 10.1186/s12931-024-02667-8

**Published:** 2024-01-12

**Authors:** Lisa Zhang, Natalie Kelly, Kimberly M Shontz, Cynthia L. Hill, Jacob T. Stack, Jazmin Calyeca, Laura Matrka, Audrey Miller, Susan D Reynolds, Tendy Chiang

**Affiliations:** 1 Department of Otolaryngology-Head and Neck Surgery, The Ohio State Wexner Medical Center, Columbus, OH USA; 2 The Ohio State University College of Medicine, Columbus, OH USA; 3 Department of Otolaryngology, Nationwide Children’s Hospital, 555 S. 18th St, Suite 2A, Columbus, OH 43205 USA; 4 Center for Regenerative Medicine, Abigail Wexner Research Institute at Nationwide Children’s Hospital, Columbus, OH USA; 5 Center for Perinatal Research, Abigail Wexner Research Institute at Nationwide Children’s Hospital, Columbus, OH USA; 6 Comprehensive Center for Bronchopulmonary Dysplasia, Department of Pediatrics, Division of Neonatology, Nationwide Children’s Hospital, Columbus, OH USA

**Keywords:** Squamous basal cell, Tracheal brushing, Host epithelium

## Abstract

**Backgorund:**

Tissue-engineered tracheal grafts (TETG) can be recellularized by the host or pre-seeded with host-derived cells. However, the impact of airway disease on the recellularization process is unknown.

**Methods:**

In this study, we determined if airway disease alters the regenerative potential of the human tracheobronchial epithelium (hTBE) obtained by brushing the tracheal mucosa during clinically-indicated bronchoscopy from 48 pediatric and six adult patients.

**Results:**

Our findings revealed that basal cell recovery and frequency did not vary by age or region. At passage 1, all samples produced enough cells to cellularize a 3.5 by 0.5 cm^2^ graft scaffold at low cell density (~ 7000 cells/cm^2^), and 43.75% could cellularize a scaffold at high cell density (~ 100,000 cells/cm^2^). At passage 2, all samples produced the number of cells required for both recellularization models. Further evaluation revealed that six pediatric samples (11%) and three (50%) adult samples contained basal cells with a squamous basal phenotype. These cells did not form a polarized epithelium or produce differentiated secretory or ciliated cells. In the pediatric population, the squamous basal cell phenotype was associated with degree of prematurity (< 28 weeks, 64% vs. 13%, *p* = 0.02), significant pulmonary history (83% vs. 34%, *p* = 0.02), specifically with bronchopulmonary dysplasia (67% vs. 19%, *p* = 0.01), and patients who underwent previous tracheostomy (67% vs. 23%, *p* = 0.03).

**Conclusions:**

In summary, screening high-risk pediatric or adult population based on clinical risk factors and laboratory findings could define appropriate candidates for airway reconstruction with tracheal scaffolds.

*Level of evidence.* Level III Cohort study.

**Graphical Abstract:**

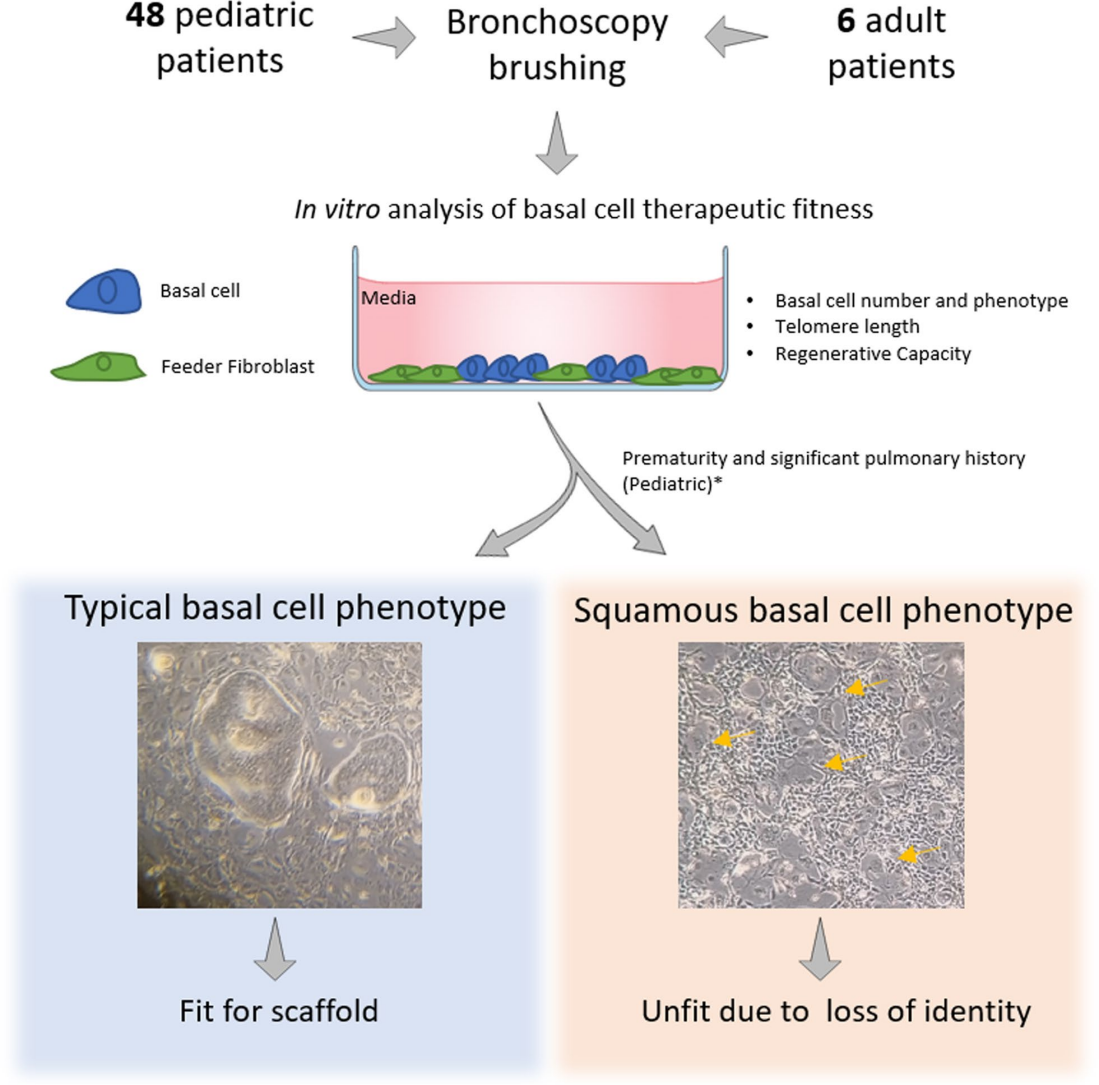

## Background

Laryngotracheal stenosis (LTS), defined as soft tissue narrowing of the proximal airway, currently has an estimated incidence of 0.6-2% [1–3]. The causes of LTS can be multifactorial in nature. In the pediatric and adult populations, acquired stenosis is most commonly secondary to prolonged intubation, tracheostomy, systemic disease, trauma, infection, or idiopathic causes [4, 5]. Surgical management of LTS can range from tracheostomy to endoscopic and open approaches [1]. Despite advancements in endoscopic and open techniques, there are significant limits to current clinical approaches.

Long-segment tracheal defects are defined as > 50% in adults and > 30% in children [6]. These defects cannot be repaired primarily because they require replacement tissue that is not currently available. A myriad of bioengineering approaches have created scaffolds that recapitulate the biological and mechanical properties of the native trachea [7, 8]. The success of these scaffolds is dependent in part on rapid epithelialization, which restores mucociliary clearance and prevents infection and inflammation [9]. However, the optimal method for reepithelialization is unknown.

We demonstrated that host-derived basal cells regenerate a surface airway epithelium (SAE) on a partially decellularized tracheal scaffold [10]. This process requires 7–14 days and could be accelerated by pre-seeding the scaffold with patient-derived cells. Ex vivo methods of cell seeding include the use of induced pluripotent stem cell-derived basal cells and primary airway stem cells (basal cells) [11]. Although both cell types can be expanded using current cell culture technology, the quality of the cell therapy product is likely to be dependent on the initial cell inoculum.

Patients with long-segment tracheal defects can suffer from chronic airway disease, a situation that may compromise the regenerative potential of the host’s basal cells. In particular, stenotic regions of the airway exhibit abnormal epithelial morphology, including basal cell dysplasia [12, 13]. We reported that the lifespan of the tracheobronchial basal cell is ~ 40 population doublings [14], which is consistent with the Hayflick limit [15]. Stem cell frequency decreases during wound healing and in vitro expansion, resulting in reduced regenerative potential [16, 17]. These decrements are associated with a decrease in the number of clone-forming stem cells, accumulation of cells with shortened telomeres, and suboptimal production of differentiated secretory and ciliated cells [18]. Thus, a critical question is whether tracheobronchial stem cells from patients with airway disease have sufficient regenerative potential to support the cellularization of a graft and the production of a functional epithelium. We addressed this issue by assessing the phenotype and function of basal cells that were recovered from the airways of normal donors and those with chronic lung disease.

## Methods

### Approvals

This study was approved by the institutional review boards of two tertiary-level pediatric and adult hospitals (Nationwide Children’s Hospital IRB STUDY00000847, Ohio State University 2021N0027). Pediatric and adult patients undergoing scheduled direct laryngoscopy and bronchoscopy (DLB) were voluntarily recruited. Demographics of the patients recruited were recorded.

### Tracheal airway epithelial cell isolation

Our methods are similar to those previously described [16, 18]. Briefly, endotracheal brush biopsies were collected using a cytology brush (Medical Packing Corporation, Camarillo, CA). Under direct visualization, the cytology brush was advanced and spun in a focal region of the trachea five times for approximately 10 s.

Cells were liberated from the brushes using mechanical agitation in phosphate-buffered saline (PBS) supplemented with 10 µM β-mercaptoethanol (Acros Organics, NJ), 5 mM EDTA (Thermo Fisher, Waltham, MA), and 5 mM EGTA (Thermo Fisher, Waltham, MA). Cells were pelleted by centrifugation (2500 rpm, 5 min) and resuspended in 1 mL red blood cell lysis buffer (Invitrogen/Thermo Scientific, Waltham, MA) for 5 min. Lysis was then terminated by dilution with PBS (1:10) and the cells were repelleted. Cell pellets were then resuspended in culture medium (see below).

### Tracheal airway epithelial culture timeline

Cells were cultured using the modified conditional reprogramming culture (mCRC) method [19]. Accordingly, the cells were co-cultured on a feeder layer of irradiated NIH-3T3 fibroblasts (ATCC #CRL-1658) with F-medium (F_med_) supplemented with Rho-kinase inhibitor, Y-27632 (10 µM). Cultures were supplemented with an antibiotic/antifungal cocktail, which was reduced to 50% concentration on day 2 and discontinued on day 4 of culture. Culture medium was changed on a Monday-Wednesday-Friday schedule, and the plates were monitored for basal cell colony development. Cultures were discarded if colonies were not observed on culture day 14. Successful cultures (p0) were expanded at passage 1 (p1) and cryopreserved as frozen stocks at passage 2 (p2).

### Cytospin

Cytospin preparations (20,000 cells per slide) were prepared as previously described [14, 20], fixed with 10% neutral-buffered formalin (NBF) for 10 min at room temperature, and stained for Keratin 5 (Covance Innovative Antibodies, Rabbit, 1:1000) and Keratin 14 (Thermo Scientific, Mouse, 1:500). Slides were counterstained with 1 µg/ml 4′,6-diamidino-2-phenylindole (DAPI). Images of at least 200 nucleated cells were acquired using Zeiss Imager.Z1 fluorescent microscope (Zeiss) and quantified using ImageJ software.

### Clone-forming cell frequency assay

Clone-forming Cell Frequency (CFCF) was determined using a limiting dilution method that has been previously described [21] and modified for use with airway tissues [14]. Passage one cells were distributed to wells of a 96-well plate using two-fold dilution from 100 cells/well to 1 cell/well. Six replicates were seeded for each cell input. Following 9 days of culture, cells were fixed with NBF, and stained with Giemsa stain. Wells were scored as positive or negative for the development of colonies. Linear regression analysis was used to calculate the CFCF.

### Population Doubling Level calculation

Population Doubling Level was calculated as: PDL = 3.32 (logXe – logXb), where Xe is the basal cell number obtained at passage and Xb is the basal cell number plated (https://www.atcc.org/resources/culture-guides/animal-cell-culture-guide). Cells were stained with trypan blue and counted using a hemacytometer [15].

### Relative telomere length determination

Telomere length was determined based on previously published methods [22, 23] with minor modifications [17]. Specifically, cells were recoverd using the double trypsinization method. Genomic DNA (gDNA) was purified from 50,000 cells using the DNeasy Blood & Tissue Kit (Qiagen Cat# 69506) and quantified using a Nanodrop 2000 spectrophometer (ThermoFisher, Welmington, DE). Both the telomere and 36B4 single copy gene control assays used 40 ng gDNA/reaction. Targets were amplified using previously reported standards and primers [22]. For the telomere assay, the magnesium concentration in the PowerSYBR Green Master Mix (Applied Biosystems  Cat# 4367659) was lowered to a final concentration of 0.9625 mM through the addition of 0.5 M EDTA (0.0385 µL per 20 µL reaction). Previously published cycling parameters for telomeres [23] and 36B4 [22] were used. Jurkat (ATCC) and U1301 (human T-cell leukemia cell line (Sigma Cat# 01051619) were used as short and long telomere controls, respectively. A standard curve was generated for each experiment and was used to insure a linear relationship between DNA input and telomere length. Prior to analysis, the telomere standards were incubated at 95 °C with shaking for 5 min, and immediately placed on ice. Three technical replicates were generated for each standard curve point and biological sample. Relative telomere length was calculated according to the Telomere/Single copy gene control (T/S) ratio.

### Flow cytometry

Human basal cells were resuspended at 10^7 cells/mL in BSA staining buffer (BD Pharmingen Cat# 554657). The cells were stained with APC-anti-CD151 (Biolegends Cat# 350406, 1:20) and EFlour 450-anti-CD49f (Invitrogen Ref# 48-0495-82, 1:20) for 30 min on ice. The cells were then washed with BSA staining buffer, fixed in NBF, and analyzed on a BD Pharmingen Fortessa-5 laser flow cytometer. Unstained cells served as a negative control. Flow data was analyzed using FlowJo.

### Correlation of clinical and culture phenotype

Univariate chi-squared and t-test analyses were used to compare patient demographic and clinical characteristics between those who did and did not have squamous basal cells. Clinical characteristics included prematurity; significant pulmonary, cardiac, gastrointestinal, and/or neurological history; known chromosomal abnormalities; congenital defects in the head and neck (including supraglottic, glottic, subglottic, tracheal stenosis); and history of airway surgery. Prematurity was coded as an ordinal variable per World Health Organization guidelines, with a moderate to late preterm group (gestational age between 32 and 37 weeks), a very preterm group (28 to 32 weeks), and an extremely preterm group (less than 28 weeks).

### Statistical analysis

All statistical calculations were conducted with Stata 17.0 (College Station, TX). Summary data are presented as the mean and standard deviation. Symbols represent the values from individual donors. Sample size is noted in the figure legends. Normally-distributed data were assessed by student’s t-test or 2-way ANOVA. Skewed data sets were assessed using the ROUT method and analyzed using GraphPad Prism, version 9.0.0 (GraphPad Software Inc., CA). A significance threshold of 0.05 was used. Specific analysis details for each data set and corresponding N for each experimental set are indicated in the figure legends.

### Power analysis

Power analyses were completed with our pediatric population (total sample size of 48 patients), demonstrating that with our univariate chi-squared analyses with an error probability of 0.05 and power of 0.8, we would be able to detect a large effect size (*r* = 0.49). In our adult population (total sample size of 6 patients) with similar power analyses, we would be able to detect an effect size of 0.78. In order to detect a medium effect size (*r* = 0.3), we would need a total sample size of 143 patients in each of the pediatric and adult cohorts.

## Results

### Pediatric donor characteristics

This study included 48 pediatric patients and 6 adult patients. Male pediatric donors comprised 67% (*N* = 31) of the cohort and had a mean age of 3.5 years (standard deviation, S.D., 3.7 years). The mean female pediatric donor age was 3.4 (S.D. 3.5 years). The mean age did not vary for the two groups (*p* = 0.85). Mean body mass index (BMI) percentile was 51.6 (SE = 35). Prematurity was the most common comorbidity (62%, *N* = 30). Bronchoscopy was commonly performed for recurrent croup (*N* = 8, 17%), stridor (*N* = 8, 17%), or in conjunction with other surgical procedures (*N* = 6, 13%), including adenotonsillectomy for obstructive sleep apnea (*N* = 3, 50%). Full patient demographic information for the pediatric population is presented in Table 1.

**Table 1:** Clinical characteristics of pediatric patients

	Pediatric Patients (*N* = 48)
Age (years, [SD])	3.5 years [3.6]
Sex	
Male	32 (67%)
Female	16 (33%)
Race	
White/Caucasian	37 (77%)
Black/African American	8 (17%)
Multi-racial/Other	3 (6%)
BMI percentile (mean [SD])	51.6 [35]
Prematurity (gestational age at birth)	
At term	18 (38%)
32–37 weeks (moderate/late preterm)	18 (38%)
29–32 weeks (very preterm)	4 (8%)
< 28 weeks (extremely preterm)	8 (16%)
Primary clinical indications for DLB	
Recurrent croup	8 (17%)
Stridor	8 (17%)
Part of other procedure	6 (13%)
Surveillance following airway surgery	5 (10%)
Aspiration	5 (10%)
Tracheal obstruction/lesion	4 (8%)
Supraglottic obstruction/lesion	4 (8%)
Dysphonia	4 (8%)
Subglottic obstruction/lesion	3 (6%)
Failure to extubate	1 (2%)

*DLB* direct laryngoscopy and bronchoscopy

### Adult donor characteristics

Six adult donors were recruited for this study. The cohort was 50% (*N* = 3) male, with mean age of 55 years (SE 13.2 years). 50% (*N* = 3) of the patients underwent direct laryngoscopy (DL) for subglottic stenosis and 33% (*N* = 2) for tracheal stenosis. The final patient had a normal airway and was recruited as a healthy control. Additional patient characteristics of the adult population are detailed in Table 2.

**Table 2:** Clinical characteristics of adult patients

	Adult Patients (*N* = 6)
Age (years, [SE])	55 years [13.2]
Sex	
Male	3 (50%)
Female	3 (50%)
Race	
White/Caucasian	3 (50%)
Asian	1 (17%)
Other	2 (33%)
Primary indication for DL^a^	
Subglottic stenosis	3 (50%)
Tracheal stenosis	2 (33%)
Control patient^b^	1 (16%)
History of past airway surgery	3 (50%)
Current tracheostomy	1 (17%)
Future airway interventions required	3 (50%)

^a^DL direct laryngoscopy

^b^Surgery was for Zenker’s diverticulum repair. Tracheal brushing obtained as a control patient

### Basal cell number and phenotype

**Fig. 1:**
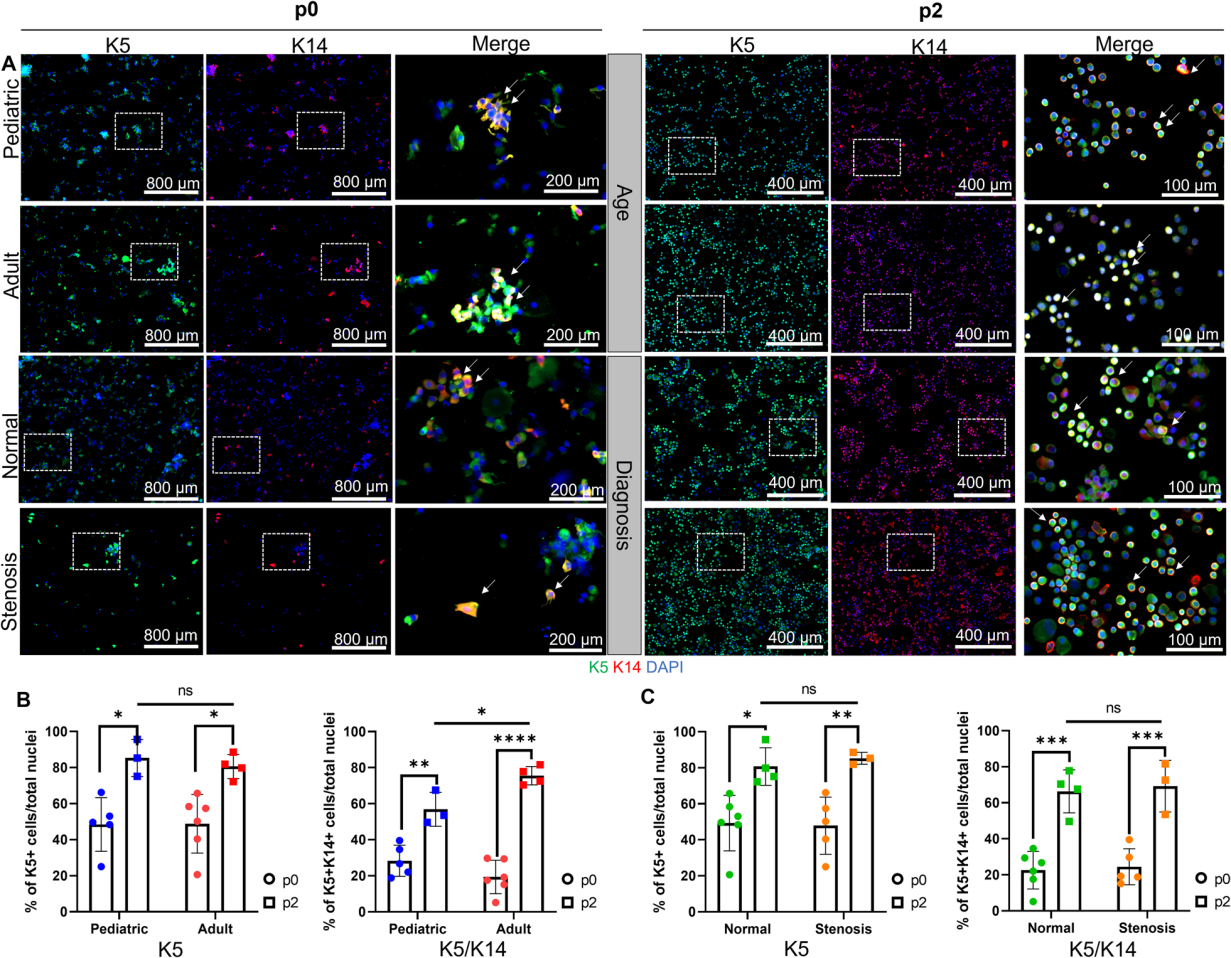
Similar basal cell yield and expansion potential in brush biopsies. **A** Representative immunostaining of cytospin-recovered cells demonstrates positive staining of basal cell markers (K5, green) and (K14, red) in pediatric (p0 *n* = 5, p2 *n* = 3) and adult (p0 *n* = 6, p2 *n* = 4) samples, after brushing from normal (p0 *n* = 6, p2 *n* = 4), and stenotic regions (p0 *n* = 5, p2 *n* = 3) at passage 0 (left panel) and passage 2 (right panel). **B** Bar graph showing the percentage of positive cells over total cell counts (DAPI, blue) count divided by age or **C** region of sample collection. Bars represent mean ± DLB: direct laryngoscopy and bronchoscopy S.D. Statistical analysis was performed using 2-way ANOVA with multiple-comparison test. *P < 0.05, **P < 0.01, ***P < 0.001, ****P < 0.0001

Cytospin slides were generated from uncultured cells and stained for a pan-basal cell marker keratin 5 (K5) and a marker of basal cell activation keratin 14 (K14) (Fig. 1A). Regardless of age (pediatric vs. adult) or region sampled (normal vs. stenosis), basal cell number (K5+) and activation status (K5/K14+) were similar (Fig. 1B). These data indicate that the brush biopsy protocol recovered similar numbers of basal cells and that these metrics did not vary by age or region (Fig. 1C).

As the brush biopsy contains a heterogenous population of epithelial cells including differentiated mucous-producing and ciliated cell types, cell isolates were cultured using the mCRC method which selects for basal cells. To determine if age or the region of airway stenosis sampling influenced basal cell expansion in vitro, cytospins were generated from passage 2 cells and stained for K5 and K14 (Fig. 1A). The percentage of basal cells increased with passage. Basal cell number (K5 + only) and activation state (K5/K14+) did not vary as a function of age (Fig. 1B) or airway region sampled (Fig. 1C).

### Regenerative capacity

**Fig. 2:**
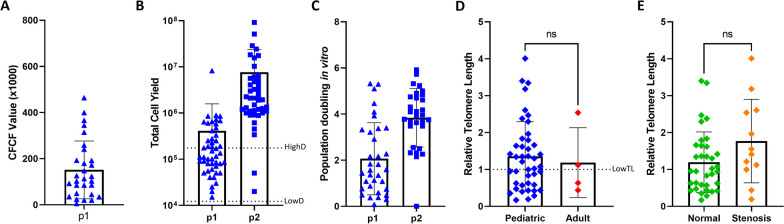
Regenerative capacity of pediatric and adult basal cells. **A** Bar graph represents the Clone-forming Cell Frequency of pediatric and adult basal cells after 1 passage in culture (*n* = 30). **B** Prediction of the number of cells recovered after 1 or 2 passages in culture (*n* = 48) able to cellularize a scaffold at low density (LowD) or high density (HighD). **C** Bar graph of the in vitro population doubling after 1 or 2 passages in culture (p1 *n* = 35, p2 *n* = 35). **D** Relative telomere length of the basal cells of pediatric (*n* = 48), and adult (*n* = 6) donors divided by **E** Normal (*n* = 59) and stenotic (*n* = 18) region. Bars represent mean ± S.D. Statistical analysis was performed using paired, 2-tailed Student’s t-test

To estimate the therapeutic potential of cells that are recovered by brush biopsy, we determined the clone forming cell frequency at passage 1. This study used the limiting dilution assay and results were reported as the Clone-forming Cell Frequency (CFCFx1000, Fig. 2A). The mean CFCF was 195 +/- 210 (*n* = 33 donors). This value was lower than the CFCFx1000 for basal cells that were isolated by digestion of explanted tracheal tissue [24]. The pediatric and adult groups and the normal or stenotic sampling sites had similar numbers of clone forming cells.

To determine if these clone forming cells could be amplified, they were passaged and cell yield was determined (Fig. 2B). These results were benchmarked against a scaffold that would be used for the reconstruction of a pediatric airway [25]. At passage 1, all samples contained sufficient cells to cellularize a 3.5 by 0.5 cm^2^ graft scaffold at low cell density (~ 7000 cells/cm^2) and 98% could cellularize a scaffold at high cell density (~ 100,000 cells/cm^2). At passage 2, all samples contained sufficient cells for both recellularization models.

To determine if these cells had reserve regenerative potential, we first calculated the number of populations doublings [15]. At passage 1, most samples underwent 0–5 population doublings (Fig. 2C). However, cell number did not increase in 13 samples. At passage 2, all samples underwent 4 population doublings. The total number of population doublings was ~ 25% of basal cell life-span [20].

Finally, we used telomere length as a surrogate for biological age [26]. DNA was recovered from cryopreserved p2 cells (48 pediatric and 5 adult subjects). Telomere length was analyzed using a modified PCR assay and is presented as the relative telomere length [17]. Since telomere length varies as a function of chronological age and has been associated with lung diseases [27], we evaluated pediatric and adult samples separately. At passage 2, the relative telomere length for pediatric samples was 2.3+/-0.42, and 41% of samples were in the short telomere range (the samples with a value </=1, Fig. 2D). Relative telomere length in adult samples was 1.6+/-0.3 and most samples were in the short telomere range (Fig. 2D). No significant differences were detected between normal or stenotic samples (Fig. 2E). Collectively, these data indicate that the airways of pediatric and adult 54 donors have sufficient regenerative capacity to recellularize an airway scaffold.

### Alteration of basal cell identity: squamous basal cells

**Fig. 3:**
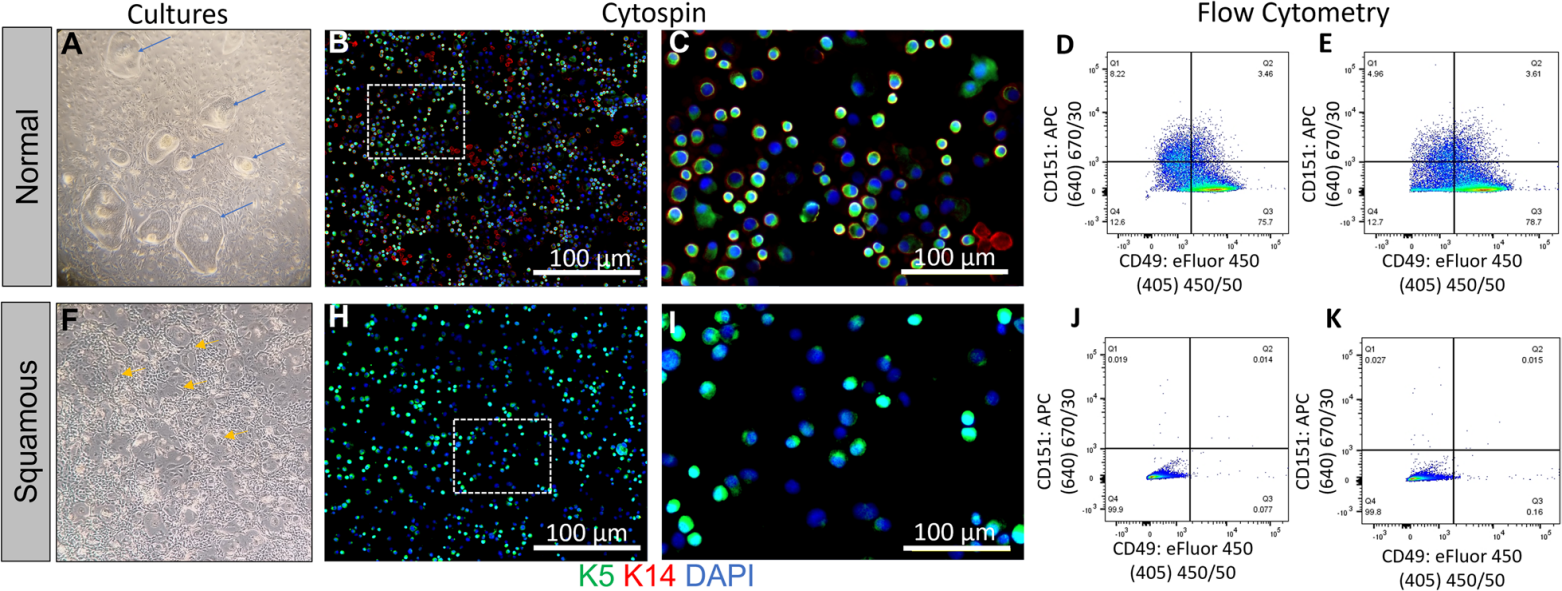
Identification of squamous basal cells. **A** Normal tracheal basal cell colonies under 10x magnification form discrete colonies with distinct edges. Representative immunofluorescence staining demonstrates high levels of K5 (green) and K14 (red), markers associated with airway epithelial stem cells, at low **B** and high **C** magnification. Representative double staining with epithelial cell markers CD151 (α-CD151-APC) and CD49f (α-CD49f-eFlour-450) in normal **D** and stenotic **E** regions. **F** Squamous metaplasia cells under 10x magnification exhibit a diffuse growth pattern and no discrete edges.Representative immunofluorescence staining demonstrates high levels of K5 (green) and K14 (red), markers associated with airway epithelial stem cells, at low **H** and high **I** magnification. Representative double staining with epithelial cell markers CD151 (α-CD151-APC) and CD49f (α-CD49f-eFlour-450) in normal **I** and stenotic **J** regions

Culture morphology was qualitatively assessed at p1. Typical basal cells (normal phenotype) formed discrete colonies on the fibroblast feeder layer that were generally spherical or ovoid in shape and had distinct edges. Normal phenotype colonies required extensive trypsin exposure to remove them from the culture plate and to dissociate the cells into a single-cell suspension for passage and handling. Most pediatric donor collections yielded basal cell colonies of typical phenotype (59 of 69 samples, 85.5%, Fig. 3A). These cells expressed high levels of K5 and K14 (Fig. 3B, C).

In contrast, squamous basal cells (Fig. 3F) were observed as morphological variants at p1. These colonies exhibited a diffuse lattice-like structure with poorly defined boundaries and expressed lower levels of K5 than typical cells (Fig. 3H, I). Both the number of K5 positive cells and the intensity of the staining were decreased relative to cells recovered from typical morphology colonies. Abnormal cells grew more rapidly than typical basal cells and were easily dislodged with trypsin. Ten cultures originating from seven pediatric patients (14.5%) contained squamous basal cell colonies at passage 1. Samples containing these abnormal cells were excluded from the previous data sets (Figs. 1, 2 and 3).

Cell phenotype was further evaluated using flow cytometry. Cells with a typical phenotype demonstrated high expression of the markers CD151 and CD49f (Fig. 3D, E), which are commonly expressed on epithelial basal cells [13]. In contrast, cells with the squamous basal cell phenotype had decreased or absent expression of these markers (Fig. 3K–J). Squamous basal cells from pediatric and adult donors exhibited similar morphology and marker expression profiles.

A meta-analysis demonstrated that the presence of squamous basal cells correlated with the degree of prematurity, with 67% (*N* = 4) from donors having a history of extreme prematurity (< 28 weeks, vs. 5% [*N* = 2], *p* = 0.001, see Table 3. Squamous basal cell isolates were also associated with significant pulmonary history (83% vs. 31%, *p* = 0.013), specifically bronchopulmonary dysplasia (67% vs. 17%, *p* = 0.006), as well as airway surgery (67% vs. 26%, *p* = 0.045), specifically tracheostomy placement (67% vs. 21%, *p* = 0.02). The presence of squamous basal cells was not associated with a history of laryngotracheal reconstruction or balloon dilation (33% vs. 10%, *p* = 0.1; 33% vs. 14%, *p* = 0.24; respectively). Subglottic stenosis as assessed by direct laryngoscopy and bronchoscopy approached significance (50% vs. 17%, *p* = 0.06).

Squamous basal cells were observed in one adult patient with subglottic stenosis and two patients with tracheal stenosis. Adult samples containing these abnormal cells were excluded from the previous data sets (Figs. 1, 2 and 3). The presence of squamous basal cells did not correlate with direct laryngoscopy findings (subglottic stenosis 50% vs. tracheal stenosis 100% vs. control 0%; *p* = 0.2) including aspiration, obstructive sleep apnea (OSA), past airway surgery, or current/previous tracheostomy (all *p* > 0.05).

**Table 3:** Squamous basal cell phenotype associations with the pediatric population

	Normal Phenotype (*N* = 42)	Squamous metaplasia (*N* = 6)	p-value
Age (years, [SD])	3.5 [3.7]	3.8 [1.2]	0.86
Sex			> 0.99
Male	22 (52%)	4 (67%)	
Female	14 (48%)	2 (13%)	
Race			0.79
White/Caucasian	32 (76%)	5 (83%)	
Black/African American	7 (17%)	1 (17%)	
Multi-racial/Other	3 (7%)	0 (0%)	
BMI percentile (mean [SE])	53 [5.3]	39 [16.6]	0.36
Prematurity			0.006**
At term	17 (40%)	1 (17%)	
32–37 weeks (moderate/late preterm)	17 (40%)	1 (17%)	
29–32 weeks (very preterm)	4 (10%)	0 (0%)	
< 28 weeks (extremely preterm)	4 (10%)	4 (67%)	
Significant pulmonary history	13 (31%)	5 (83%)	0.013**
Bronchopulmonary dysplasia	7 (17%)	4 (67%)	0.006**
Significant cardiac history	0 (0%)	1 (17%)	0.008**
History of previous airway surgery	11 (26%)	4 (67%)	0.045**
Previous tracheostomy	9 (21%)	4 (67%)	0.02**
Previous laryngotracheal reconstruction	4 (10%)	2 (33%)	0.099
Previous balloon dilation	6 (14%)	2 (33%)	0.24
Findings on DLB^a^			
Subglottic stenosis	7 (17%)	3 (50%)	0.06
Tracheal stenosis	1 (2%)	1 (17%)	0.10
Laryngomalacia	4 (10%)	0 (0%)	0.43
Vocal cord nodules	3 (7%)	0 (0%)	0.50
Average Relative Telomere Length [SE]	3.0 [1.5]	3.2 [1.9]	0.95

Univariate analyses were conducted, and statistically significant predictors were calculated with *p*<0.05 and marked (**)

^a^DLB: direct laryngoscopy and bronchoscopy

## Discussion

Our study revealed that the airways of pediatric and adult donors have sufficient regenerative capacity to recellularize an airway scaffold. However, we identified squamous basal cells in a subset of donors and associated their presence with chronic lung disease and tracheostomy in the pediatric population.

The basal cell is the tissue-specific stem cell of the conducting airway epithelium [28]. Common pediatric airway diseases including bronchopulmonary dysplasia [29] and laryngotracheal stenosis [30] can result in epithelial injury from mechanical ventilation, direct trauma, infection, and inflammation. Repeated injury causes selective activation of basal cells [17] and promotes proliferation. These events can lead to terminal differentiation and squamous metaplasia [31–33].

In conditions such as chronic obstructive pulmonary disease (COPD), pathologic remodeling of the airway epithelium includes squamous metaplasia. This condition is likely to be the result of environmental exposures and a chronic inflammatory response and is thought to contribute to poor ciliary function and mucus clearance [28]. Sustained pathogeneic squamous phenotype has been associated with the pathophysiology of tracheobronchial disorders with unknwon etiology, accompanied by recurrent infections, nodules formation, and absence of normal ciliated respiratory epithelium [34]. Squamous metaplasia has also been reported in the proximal airways following intubation and tracheostomy placement. Although the clinical implications of squamous metaplasia remain unclear [13], McKeon’s group identified a squamous basal cell subtype that self-renewed and retained its phenotype across multiple passages and after transplantation into immunocompromised mice [31]. We identified similar cells in our cohort and report that they are more prevalent in pediatric patients with bronchopulmonary dysplasia, subglottic stenosis, and those who underwent tracheostomy placement.

The association between bronchopulmonary dysplasia and squamous basal cells requires further study. The observed correlations may be a consequence of direct trauma to the airway during intubation and surgery in combination with free radical changes from prolonged intubation. It is also possible that the squamous metaplasia phenotype is congenitally predisposed in certain pediatric patients, although the relationship was not clearly demonstrated [13]. If substantiated, this predisposition would explain why premature patients have significantly higher rates of squamous basal cells compared to their full-term peers [35].

## Future directions

The rise of tissue engineering has provided promising techniques with the intention of creating viable tracheal replacement grafts [8, 36, 37]. Successful grafting not only requires an optimal cell source and scaffold for integration but also the appropriate cellular microenvironment for complete epithelialization [8]. Although our data indicate that brush biopsy cells have significant therapeutic potential, a subset of samples contained squamous basal cells that do not regenerate the epithelium. Further studies could determine if cell rejuvenation methods would improve the performance of samples with low-performance metrics.

## Study limitations

We recognize that our understanding of the squamous basal cell phenotype is limited by sample size and that the study may be underpowered to detect smaller effect sizes, particularly those related to tracheal stenosis. We also acknowledge that our cross-sectional study design could be enhanced by a longitudinal study. Finally, we understand that our sampling method may have underestimated the frequency of squamous basal cells in pediatric and adult airways.

## Conclusions

Our data suggest that screening high-risk pediatric or adult population based on clinical risk factors and laboratory findings could define appropriate candidates for airway reconstruction with tracheal scaffolds.

## Data Availability

Not applicable.
